# Permeation Behaviors of MFI Zeolite Membranes Activated by Rapid Ozonation

**DOI:** 10.3390/membranes16040122

**Published:** 2026-03-31

**Authors:** Zhenming Yi, Zilin Pan, Feng Ye, Shuanshi Fan, Xuemei Lang, Yanhong Wang, Gang Li

**Affiliations:** School of Chemistry and Chemical Engineering, South China University of Technology, Guangzhou 510641, China

**Keywords:** MFI zeolite membrane, ozonation, activation, H_2_ separation, pervaporation

## Abstract

Conventional high-temperature calcination for activating MFI zeolite membranes is energy-intensive and prone to inducing defects. Here, we demonstrate that a rapid ozonation treatment at 200 °C for only 1 h effectively decomposes organic templates while preserving membrane integrity. The resulting membrane exhibits H_2_/CH_4_ and H_2_/N_2_ ideal selectivities of 10.3 and 6.5, respectively, at room temperature, with C_3_H_8_ and SF_6_ permeances below the detection limit. These results confirm a dense, defect-minimized architecture and good molecular sieving performance of the zeolite membrane. In contrast, extending ozonation to 48 h leads to defect formation and a marked reduction in selectivity. For H_2_/CH_4_ mixture separation, the membrane achieves a selectivity of 23.8 at 100 °C, which is highly competitive among reported MFI membranes. In isopropanol dehydration, it achieves a water flux of 2.3 kg·m^−2^·h^−1^ and a separation factor of 3278 at 70 °C with a 10 wt% water feed, while maintaining >99.5 wt% water content in the permeate over a broad operating temperature range (30–70 °C). This work establishes rapid ozonation as a scalable, energy-efficient activation method for high-performance MFI zeolite membranes in both gas and liquid separations.

## 1. Introduction

Membrane separation technology, known for its exceptional energy efficiency, has become a pivotal tool for enabling highly energy-conserving chemical processes [1,2,3]. Among various membrane materials, zeolite membranes stand out as particularly promising candidates for molecular-scale separations, owing to their uniform, sub-nanometer pores and exceptional thermal and chemical stability. Within this category, MFI-type zeolite membranes (e.g., Silicalite-1, ZSM-5) have attracted extensive research interest. Their well-defined pore system (~0.55 nm) enables precise size and shape-selective separation of molecules such as light hydrocarbons [4,5,6], xylene isomers [7,8,9], and alcohols [10,11,12], highlighting their strong potential for key industrial applications.

However, the transition of MFI zeolite membranes from laboratory prototypes to industrial implementation faces a major bottleneck in membrane activation—a critical step for removing organic structure-directing agents (OSDAs) from the zeolitic pores. Conventional activation relies on high-temperature calcination (400–600 °C) in an air or oxygen atmosphere, typically employing very slow heating and cooling rates (0.5–1 °C·min^−1^) [13,14,15]. This approach is not only time-consuming and energy-intensive but also induces considerable thermal stress within the membrane [16,17], which can promote the formation of grain-boundary defects and ultimately lead to a significant loss of membrane selectivity [7,18,19]. Therefore, improving the activation process is crucial for advancing the practical application of zeolite membranes.

Ozonation has been proposed as a promising alternative to conventional high-temperature calcination for zeolite membrane activation. As a potent oxidant, ozone is capable of decomposing OSDAs under significantly lower temperatures, thereby mitigating thermal stress and preserving membrane integrity [7,19,20,21]. Previous studies have confirmed that low-temperature ozonation can yield various zeolite membranes with high separation performance [7,20,22,23,24]. For example, Park et al. [7] compared the effects of high-temperature calcination, rapid thermal processing, and low-temperature ozonation on the microstructure and separation performance of MFI zeolite membranes. They found that the former two methods introduced defect cracks with widths of 20.2 nm and 4.8 nm, respectively, whereas the membrane remained nearly crack-free after ozonation. Consequently, the p-/o-xylene separation factor of the ozonated MFI membrane reached 2000, far exceeding the values achieved by high-temperature calcination (5.8) and rapid thermal processing (55.5). Wang et al. [22] reported that DDR membranes activated in an O_3_/O_2_ atmosphere at 200 °C produced high CO_2_/CH_4_ selectivities, while activation in air at 700 °C led to severe cracking. However, existing ozonation activation protocols typically feature prolonged durations, particularly for the small-pore zeolite membranes [22,23,25,26], hindering large-scale industrial implementation.

Given the size compatibility between the MFI zeolite pore diameter (~0.55 nm) and the ozone molecule (kinetic diameter: 0.46 nm [23], ozone diffusion into MFI membranes is expected to be more efficient compared to small-pore zeolites (e.g., CHA and DDR types). This suggests that the ozonation activation time could be substantially shortened for MFI membranes. Nevertheless, only limited research has explored the fabrication and permeation behavior of MFI zeolite membranes activated via rapid ozonation [19,20]. Heng et al. [20] reported that a 0.5 h treatment at 200 °C could activate a 2 μm-thick MFI membrane, but detailed gas separation performance was not examined. Tan et al. [19] achieved 87% template removal for MFI zeolite membrane after 8 h of ozonation, with reduced defects and enhanced selectivity, indicating that further reduction in activation time may be feasible. Given the underexplored potential of rapid ozonation for MFI membranes, studies are needed to minimize processing time without compromising their structural integrity and separation performance.

In this work, we employed a rapid ozonation strategy to activate MFI zeolite membranes, aiming to dramatically reduce the treatment time to only 1 h while maintaining or even enhancing their separation characteristics. The performance of the resulting membranes was evaluated in both gas separation (H_2_/CH_4_) and pervaporation (isopropanol dehydration). Our findings demonstrate that rapid ozonation not only simplifies membrane fabrication but also delivers competitive separation performance, offering a potential route toward scalable and energy-efficient manufacturing of high-quality zeolite membranes.

## 2. Materials and Methods

### 2.1. Preparation of MFI Zeolite Seeds

Tetraethyl orthosilicate (TEOS) (98 wt%, Aladdin, Shanghai, China) was dropwise added into a tetrapropylammonium hydroxide (TPAOH) solution (25 wt%, Macklin, Shanghai, China) to obtain a precursor solution with a molar composition of 1.0 TEOS: 0.3 TPAOH: 10 H_2_O. After being stirred at room temperature for 12 h, the mixture was then transferred to a Teflon-lined autoclave and subjected to hydrothermal crystallization at 115 °C for 40 h. The resulting solid was washed with deionized water to neutral pH, followed by centrifugation and drying, yielding MFI zeolite seeds.

### 2.2. Preparation of MFI Zeolite Membranes

MFI zeolite membranes were fabricated through a secondary growth approach. A uniform seed layer was first deposited onto a porous α-Al_2_O_3_ disk support (25 mm in diameter, average pore size of 200 nm) via vacuum filtration of a 0.1 wt% MFI-type zeolite seed suspension. The seeded support was then placed vertically in a Teflon-lined stainless-steel autoclave, which contained a clear precursor solution with a molar composition of 1.0 TEOS:0.2 TPAOH:100 H_2_O. Hydrothermal crystallization was conducted in an oven preheated at 150 °C for 24 h under static conditions. After synthesis, the autoclave was cooled to room temperature using flowing tap water. The resulting membrane and powder collected at the bottom of the autoclave were then thoroughly rinsed with deionized water and dried at 80 °C. Finally, the membrane and zeolite powder were activated in a tubular furnace under a continuous flow of an O_3_/O_2_ mixture (1000 mL·min^−1^; ozone concentration: 110 mg·L^−1^). The activation procedure involved heating to 200 °C at a rate of 0.1 °C·min^−1^, holding for 1 or 48 h, and cooling to room temperature at the same rate, thereby decomposing the OSDA and opening the zeolitic pores.

### 2.3. Characterization

Scanning Electron Microscopy (SEM) imaging was performed on a COXEM EM-30PLUS instrument (COXEM Co., Ltd., Daejeon, Korea) operated at an accelerating voltage of 15 kV. X-ray diffraction (XRD) analysis was conducted using a Bruker D8 ADVANCE (Bruker AXS, Karlsruhe, Germany) diffractometer equipped with Cu Kα radiation (λ = 0.15418 nm). The XRD patterns were recorded over a 2*θ* range of 5–60° at a scanning rate of 0.2 °·s^−1^. N_2_ adsorption–desorption isotherms were measured at −196 °C using a Micromeritics ASAP 2460 (Micromeritics, Norcross, GA, USA), and samples were outgassed overnight at 200 °C prior to the test.

### 2.4. Permeation Performance Evaluation

The permeation performance of MFI zeolite membranes was evaluated through both gas separation and pervaporation dehydration. Single-gas permeation experiments were performed at a constant feed pressure of 0.2 MPa across 25–200 °C. For H_2_/CH_4_ binary mixture separation, the tests were performed at 25–200 °C with the feed pressure varied between 0.2 and 0.5 MPa and the permeate side maintained at atmospheric pressure. The feed gas mixture was supplied at 100 mL·min^−1^, while the permeate side was swept with Ar at 10 mL·min^−1^ to deliver the permeated stream into an online gas chromatograph for composition analysis. Gas permeance (*P_i_*), ideal selectivity ($S_{i/j}^{single}$) for single gases, and separation selectivity ($S_{i/j}^{binary}$) for binary mixtures were calculated using the following equations:

(1) $$P_{i}=\frac{Q}{\Delta p\cdot A}$$

(2) $$S_{i/j}^{single}=\frac{P_{i}}{P_{j}}$$

(3) $$S_{i/j}^{binary}=\frac{P_{i}}{P_{j}}$$

where *Q* is the molar flow rate on the permeate side, Δ*p* is the transmembrane pressure drop, *A* is the effective permeation area of the membrane, and *P_i_* and *P_j_* are the permeances of components *i* and *j*, respectively.

Pervaporation performance was evaluated using a custom-built setup. The membrane was sealed with an O-ring and immersed in the feed solution. The feed side was maintained at atmospheric pressure with temperatures ranging from 30 to 70 °C. The permeate side was evacuated to approximately at 10 Pa by a vacuum pump, and the permeated vapor was condensed and collected in a liquid-nitrogen cold trap. Permeate composition was analyzed by gas chromatography. The permeation flux (*J*) and separation factor ($\alpha_{i/j}$) were calculated as follows:

(4) $$J=\frac{m}{A\cdot t}$$

(5) $$\alpha_{i/j}=\frac{Y_{i}/Y_{j}}{X_{i}/X_{j}}$$

where *m* is the mass of the collected permeate, *A* is the effective membrane area, *t* is the pervaporation time, and *Y_i_*, *Y_j_* and *X_i_*, *X_j_* are the mass fractions of components *i* and *j* in the permeate and feed, respectively.

## 3. Results and Discussion

### 3.1. Fabrication of MFI Zeolite Membranes

**Figure 1:**
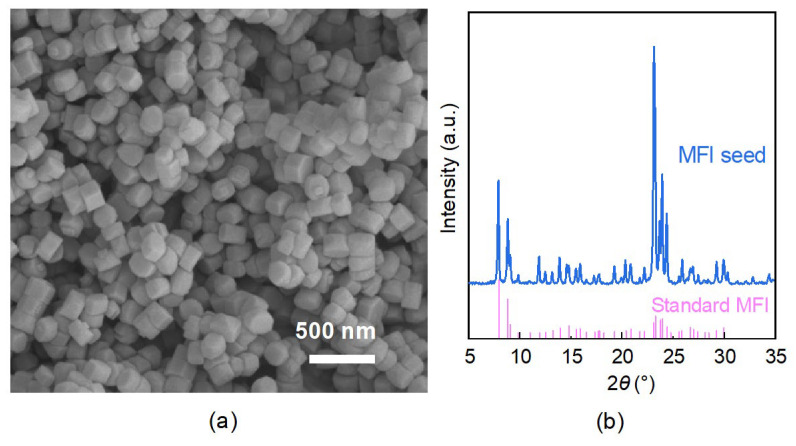
(**a**) SEM image and (**b**) XRD pattern of MFI zeolite seeds.

**Figure 2:**
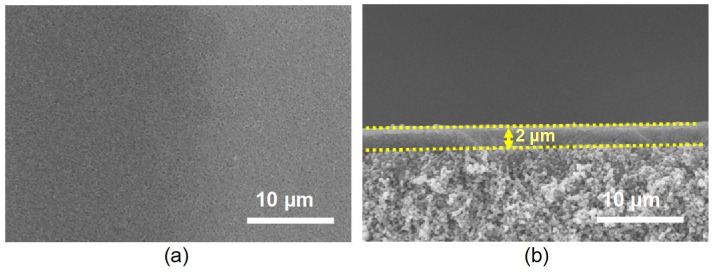
(**a**) Top-view and (**b**) cross-sectional SEM images of the seeded support.

Figure 1a shows the SEM image of the synthesized MFI zeolite seeds, revealing uniform particle morphology with an average size of approximately 200 nm. The XRD pattern (Figure 1b) confirms the MFI topology, showing characteristic peaks in good agreement with the standard diffraction data [5,27]. By depositing these seeds onto the α-alumina support via vacuum filtration, a uniform and continuous seed layer with a thickness of approximately 2 μm was obtained (Figure 2a,b). The high-quality seed layer provides abundant nucleation sites on the support surface, thereby effectively promoting zeolite crystal intergrowth during secondary growth and resulting in a highly dense membrane.

**Figure 3:**
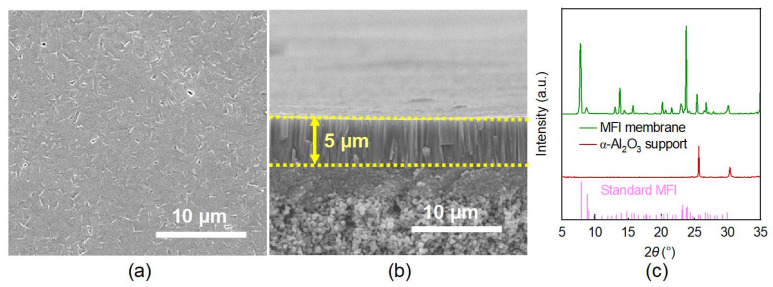
(**a**) Top-view and (**b**) cross-sectional SEM images, and (**c**) the corresponding XRD pattern of the as-prepared MFI zeolite membrane.

After hydrothermal synthesis, a dense and continuous MFI zeolite membrane was obtained. As shown in Figure 3a, the membrane exhibited well-intergrown crystals without visible pinholes or cracks, with a uniform thickness of approximately 5 μm (Figure 3b). The XRD pattern further confirms the pure MFI topology, with all characteristic peaks matching the standard reference [5,27]. Importantly, prior to activation, the He permeance through the as-synthesized membrane was below the detection limit (≤1 × 10^−12^ mol·m^−2^·s^−1^·Pa^−1^), indicating the formation of a highly dense structure with negligible grain boundary defects.

### 3.2. Single-Gas Permeation

**Figure 4:**
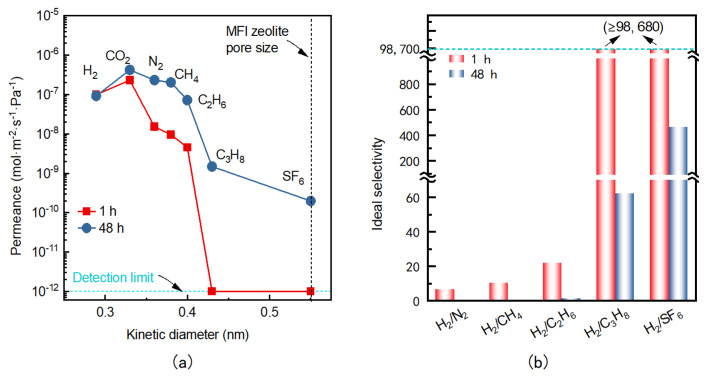
Single-gas permeation performance at room temperature for MFI zeolite membranes activated for 1 and 48 h: (**a**) gas permeance and (**b**) ideal selectivity.

Figure 4 presents the room-temperature single-gas permeation performance of MFI zeolite membranes after 1 h and 48 h ozonation activation. After 1 h activation, the membrane exhibited relatively low H_2_ and CO_2_ permeances of 9.9 × 10^−8^ and 2.3 × 10^−7^ mol·m^−2^·s^−1^·Pa^−1^, respectively. The higher permeance of CO_2_ compared to H_2_, despite its larger kinetic diameter (0.33 nm vs. 0.289 nm), can be attributed to stronger adsorption of CO_2_ within the zeolitic pores. Apart from CO_2_, gas permeances decreased with increasing kinetic diameter, giving ideal selectivities of H_2_/N_2_ = 6.5, H_2_/CH_4_ = 10.3, and H_2_/C_2_H_6_ = 21.8. Molecules with kinetic diameters approaching the MFI pore size (C_3_H_8_: 0.43 nm; SF_6_: 0.55 nm) showed permeances below the detection limit (≤1 × 10^−12^ mol·m^−2^·s^−1^·Pa^−1^), leading to extremely high ideal selectivities (both H_2_/C_3_H_8_ and H_2_/SF_6_ ≥ 98,680). These results demonstrate an excellent molecular-sieving ability and a nearly defect-free membrane structure. Furthermore, MFI zeolite membranes prepared under identical conditions exhibited highly consistent permeation behaviors, demonstrating good reproducibility of the rapid ozonation activation process. Extending the activation to 48 h led to a marked change in permeation behavior. While H_2_ and CO_2_ permeances increased only slightly (to 1 × 10^−7^ and 4.2 × 10^−7^ mol·m^−2^·s^−1^·Pa^−1^, respectively), the permeances of larger molecules rose substantially, especially for C_3_H_8_ and SF_6_. Consequently, the ideal selectivities of H_2_ against N_2_, CH_4_, and C_2_H_6_ all decreased compared with those of the rapidly activated zeolite membranes. Most notably, the H_2_/C_3_H_8_ and H_2_/SF_6_ selectivities sharply dropped to 62.2 and 465.2, approximately 2–3 orders of magnitude below their counterparts after the short activation process. This pronounced decline indicates that prolonged ozonation promotes the formation of non-selective grain-boundary defects rather than merely opening the zeolitic pores. Therefore, from both defect-control and energy-efficiency perspectives, rapid ozonation is a preferable approach for fabricating high-performance MFI zeolite membranes.

**Figure 5:**
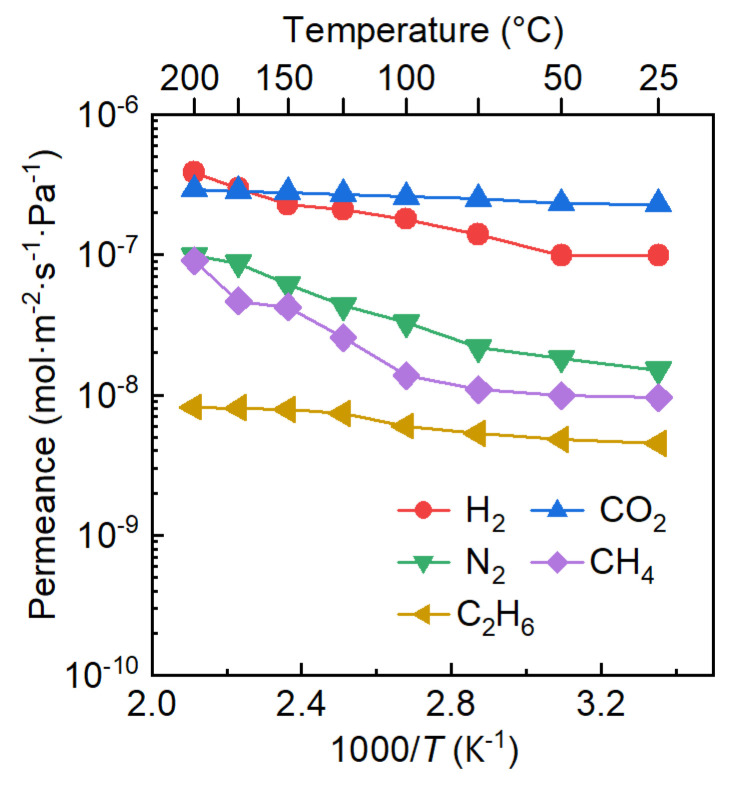
Temperature dependence of single-gas permeation through MFI zeolite membranes activated by ozonation at 200 °C for 1 h.

Figure 5 shows the temperature-dependent single-gas permeance of the MFI membrane activated for 1 h. All tested gases (H_2_, CO_2_, N_2_, CH_4_, and C_2_H_6_) exhibited increased permeance with temperature, indicating their permeation were governed by an activated diffusion mechanism. For instance, the H_2_ permeance increased from 9.9 × 10^−8^ mol·m^−2^·s^−1^·Pa^−1^ at 25 °C to 3.9 × 10^−7^ mol·m^−2^·s^−1^·Pa^−1^ at 200 °C, which were close to most of the state-of-the-art zeolite membranes. In contrast, the CO_2_ permeance was primarily reportedly decreased with increasing temperature, as its permeation was dominated by a surface diffusion mechanism [28,29,30]. This observation indicates the membrane activated by rapidly ozonation possible have a different pore structure, possibly due to the formation of a smaller average pore size for the membrane.

The temperature dependence of gas permeance follows an Arrhenius-type relationship:

(6) $$P=k\cdot exp\left(-\frac{E_{a}}{RT}\right)$$

where *P* is the permeance, *k* is the pre-exponential factor, *R* is the ideal gas constant, and *T* is the temperature, and *E_a_* is the apparent activation energy for permeation. Thus, *E_a_* for gas permeation through an MFI zeolite membrane can be obtained by fitting the natural logarithm of permeance (ln *P*) versus the reciprocal of temperature (1/*T*). The activation energy *E_a_* can be further expressed as the difference between the activation energy for diffusion (*E_d_*) and the heat of adsorption (*E_s_*):

*E_a_* = *E_d_* − *E_s_* (7)

**Figure 6:**
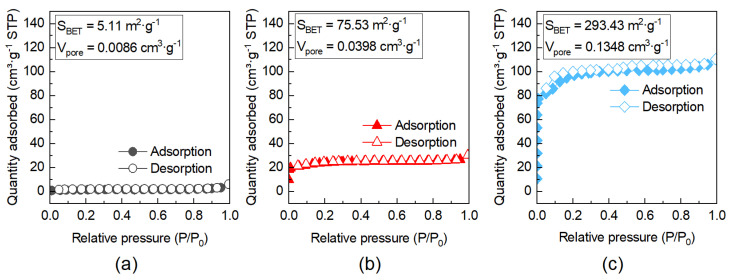
N_2_ adsorption–desorption isotherm of MFI zeolite powders: (**a**) as-synthesized, (**b**) activated by 1 h ozonation, and (**c**) activated by 48 h ozonation.

Table 1 presents the calculated activation energies (*Eₐ*) for the permeation of various gases through the MFI zeolite membrane activated by ozonation at 200 °C for 1 h. The activation energy for H_2_ permeation was determined to be 9.27 kJ·mol^−1^, which is notably higher than values reported for conventional zeolite membranes [31,32,33,34,35]. This result suggests that the MFI membrane activated via rapid ozonation likely possesses a relatively smaller average pore size, likely due to a reduction in grain-boundary defects and/or incomplete removal of the OSDA [19]. The *Eₐ* for CO_2_ was only 1.68 kJ·mol^−1^, lower than that for H_2_. Although the diffusion activation energy (*E_d_*) for CO_2_ is greater than that for H_2_ owing to its larger molecular size, the adsorption energy (*E_s_*) for CO_2_ is substantially higher because of its stronger affinity with the membrane. The resulting larger negative contribution from *E_s_* leads to a lower overall *Eₐ* for CO_2_. It is noteworthy that most previously reported MFI zeolite membranes exhibit negative activation energies for CO_2_ permeation, often attributed to a surface-diffusion mechanism [30,33,34]; the positive value observed here further supports the inference of a smaller average pore size in the present membrane. Furthermore, the activation energies for N_2_ and CH_4_ increased to 13.50 and 15.24 kJ·mol^−1^, respectively, primarily due to their larger molecular dimensions. In contrast, C_2_H_6_ showed a relatively low *Eₐ* of 4.59 kJ·mol^−1^, which can be possibly ascribed to its stronger adsorption within the membrane [36]. Furthermore, N_2_ physisorption characterization (Figure 6) revealed that the BET surface area and pore volume of the zeolite powders activated for 1 h ozonation were 75.53 m^2^·g^−1^ and 0.0398 cm^3^·g^−1^, respectively, higher than those of the as-synthesized sample (5.11 m^2^·g^−1^ and 0.0086 cm^3^·g^−1^) but lower than those of a sample activated for 48 h ozonation (293.43 m^2^·g^−1^ and 0.1348 cm^3^·g^−1^), suggesting only a portion of the OSDAs was removed during rapid activation. For zeolite membranes, complete OSDA removal is even more challenging due to the longer diffusion pathway for ozone, which much more likely results in only partial template removal. Collectively, these results support the conclusion that the MFI zeolite membrane activated by rapid ozonation likely has a small average pore size, highlighting its potential for separating small-molecule gas mixtures.

### 3.3. H_2_/CH_4_ Mixture Separation

**Figure 7:**
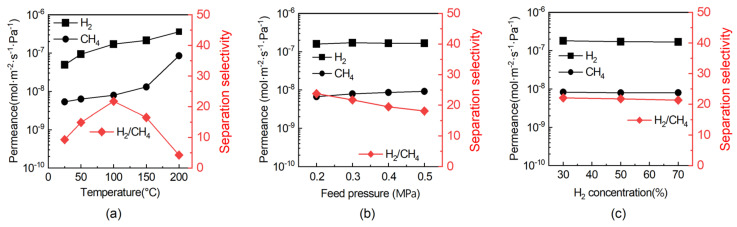
Permeation performance of MFI membrane activated at 200 °C for 1 h for separating H_2_/CH_4_ mixture as a function of (**a**) temperature, (**b**) feed pressure and (**c**) H_2_ concentration in feed stream. Testing conditions: (**a**) feed pressure: 0.3 MPa, feed composition: 50/50 vol% H_2_/CH_4_; (**b**) feed temperature: 100 °C, feed composition: 50/50 vol% H_2_/CH_4_; (**c**) feed temperature: 100 °C, feed pressure: 0.3 MPa.

The MFI membrane activated via rapid ozonation was evaluated for H_2_/CH_4_ mixture separation. The separation performance under various operating conditions is presented in Figure 7. Figure 7a shows the effect of temperature on the separation of an equimolar H_2_/CH_4_ mixture. In the mixed-gas test, the H_2_ permeance was lower than that measured in the single-gas experiment, which was commonly observed in mixed-gas permeation [25,36,37,38]. This reduction can be attributed to the preferential adsorption of CH_4_ molecules within the membrane pores, which hinders the transport of H_2_ through the membrane. As the temperature increased, the permeances of both gases increased. Below 100 °C, CH_4_ permeance increased more slowly than that of H_2_, whereas above 100 °C, it rose more rapidly. This behavior led to a maximum H_2_/CH_4_ separation selectivity of about 21.5 at 100 °C. This selectivity exceeds most reported values for MFI membranes (see Table 2), demonstrating the efficacy of the rapid-ozonation activation strategy. Figure 7b illustrates the effect of feed pressure on membrane performance using an equimolar H_2_/CH_4_ mixture at 100 °C. At 100 °C, the H_2_ permeance in the mixture was 1.6 × 10^−7^ mol·m^−2^·s^−1^·Pa^−1^, lower than that under single-gas conditions, which can be attributed to adsorption of CH_4_ within the zeolite pores that partially hinders H_2_ transport. As the feed pressure increased from 0.2 to 0.5 MPa, the H_2_ permeance remained nearly constant at approximately 1.6 × 10^−7^ mol·m^−2^·s^−1^·Pa^−1^, while the CH_4_ permeance showed a slight increase. Consequently, the H_2_/CH_4_ separation selectivity declined moderately from 23.8 to 18.1 over this pressure range. Figure 7c depicts the influence of feed composition on separation performance at 100 °C. Both H_2_ and CH_4_ permeances, as well as the H_2_/CH_4_ separation selectivity, remained essentially unchanged across different feed compositions. This behavior can be attributed to gas transport governed mainly by a size-exclusion effect under the tested conditions, with minimal influence from competitive adsorption or surface diffusion, demonstrating robust membrane performance under varying gas-mixture conditions.

### 3.4. Isopropanol Dehydration

**Figure 8:**
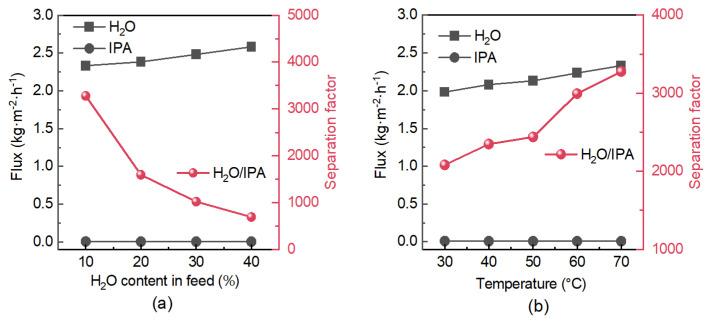
Pervaporation performance of the rapidly activated zeolite membrane for H_2_O/IPA mixture dehydration: (**a**) Water flux and separation factor as a function of water content in feed at 70 °C. (**b**) Water flux and separation factor as a function of operating temperature for a feed containing 10 wt% water.

Figure 8a shows the pervaporation performance of the rapidly activated membrane for dehydrating H_2_O/IPA mixtures as a function of water content in the feed. As the water concentration in the feed increased from 10 to 40 wt%, the water permeation flux through the membrane increased gradually from approximately 2.3 to 2.6 kg·m^−2^·h^−1^, while the IPA flux decreased slightly from 0.0064 to 0.0056 kg·m^−2^·h^−1^ under the same conditions. This behavior can be attributed to the increased chemical potential gradient for water and the relatively decreased driving force for IPA permeation at higher water concentrations. Correspondingly, once the feed water content exceeded 10 wt%, the water content in the permeate not only remained consistently above 99.7 wt% but also increased slightly with further increases in feed concentration. A maximum separation factor of 3278 was achieved with a 10/90 wt% H_2_O/IPA feed at 70 °C.

Figure 8b illustrates the temperature-dependent performance of the membrane for dehydrating a 10/90 wt% H_2_O/IPA mixture. With increasing temperature from 30 to 70 °C, the water flux rose from 2.0 to 2.3 kg·m^−2^·h^−1^, whereas the IPA flux declined from 0.0086 to 0.0064 kg·m^−2^·h^−1^. This contrasting trend reinforces the dominance of an activated diffusion mechanism for water transport, while the suppression of IPA permeation suggests competitive adsorption effects, wherein enhanced water occupancy of the pores at elevated temperatures further hinders IPA diffusion. As a result, the permeate water content remained above 99.5 wt% across the temperature range, and the separation factor increased correspondingly with temperature.

When benchmarked against conventional and advanced zeolite membranes, the performance of the short-term activated MFI membrane is notably competitive. For instance, typical LTA-type zeolite membranes report water fluxes in the range of 1–2 kg·m^−2^·h^−1^ with separation factors often exceeding 10,000 for a 10/90 wt% H_2_O/IPA mixture at 70–80 °C [42,43,44,45]. While the separation factor of the present MFI membrane (3278) is lower than that of high-selectivity LTA membranes, its water flux remains comparable while offering significantly greater framework stability. Moreover, compared with other reported MFI membranes [11,12,46], which frequently suffer from low water flux, the short-time activation strategy provides a straightforward route to improved dehydration performance. The robust separation behavior under high IPA concentrations and varying temperatures positions this membrane favorably among state-of-the-art zeolite membranes designed for organic-solvent dehydration.

## 4. Conclusions

In this work, we successfully implemented a rapid ozonation protocol (1 h at 200 °C) to activate MFI zeolite membranes, circumventing the drawbacks of traditional high-temperature calcination. The resulting membranes possess a highly intergrown, defect-minimized structure, as confirmed by SEM, XRD, and gas permeation analyses. Their exceptional molecular sieving ability (H_2_/C_3_H_8_ and H_2_/SF_6_ ideal selectivities ≥ 98,680) underscores the preservation of intrinsic zeolitic porosity almost without non-selective pathways. Prolonged ozonation (48 h), however, introduces grain-boundary defects that severely degrade selectivity, highlighting the advantage of short-duration treatment. The membrane exhibits outstanding performance in both gas and liquid-phase separations. In H_2_/CH_4_ mixture separation, it achieves a selectivity of approximately 23.8 at 100 °C under equimolar feed conditions. In IPA dehydration via pervaporation, it combines a competitive water flux (2.3 kg·m^−2^·h^−1^) with a high separation factor (3278) for a 10/90 wt% H_2_O/IPA mixture. Importantly, the rapid ozonation approach significantly reduces energy consumption and processing time, suggesting a potentially scalable route for zeolite membrane manufacturing. This work thus provides a promising strategy to enhance both the performance and manufacturing efficiency of MFI zeolite membranes for advanced separation processes.

## Figures and Tables

**Table 1:** Activation energies for gas permeation through the MFI zeolite membrane activated by ozonation at 200 °C for 1 h.

Gas	H_2_	CO_2_	N_2_	CH_4_	C_2_H_6_
*E_a_*/(kJ·mol^−1^)	9.27	1.68	13.50	15.24	4.59

**Table 2:** Comparison in gas separation performance of MFI-type zeolite membranes.

Activation Condition ^a^	Test Temperature (°C) ^a^	H_2_ Permeance (mol∙m^−2^∙s^−1^∙Pa^−1^)	H_2_/CH_4_ Ideal Selectivity	Reference
H_2_, 310 °C, 8 h	25	8.8 × 10^−9^	3.4	[31]
O_2_, 450 °C, 6 h	30	1.6 × 10^−6^	0.57	[4]
N_2_/O_2_, 400 °C,16 h	25	2.5 × 10^−7^	0.63	[28]
Air, 480 °C, 4 h	25	6.5 × 10^−6^	5.8	[39]
Air, 400 °C, 6 h	25	7.3 × 10^−6^	1.48	[40]
Air, 550 °C, 16 h	200	1.5 × 10^−7^	2.15	[41]
Air, 450 °C, 8 h	25	1.4 × 10^−6^	1.17	[19]
O_3_/O_2_, 200 °C, 8 h	25	1.0 × 10^−6^	1.13	[19]
O_3_/O_2_, 200 °C, 12 h	25	2.25 × 10^−6^	1.0	[8]
O_3_/O_2_, 230 °C, 48 h	25	5.0 × 10^−7^	2.5	[27]
O_3_/O_2_, 200 °C, 1 h	RT ^b^	9.9 × 10^−8^	10.3	This work
O_3_/O_2_, 200 °C, 1 h	100	1.6 × 10^−7^	23.8 ^c^	This work

^a^ T_c_ (°C) ≈ T_k_(K) − 273 K; ^b^ Room temperature.; ^c^ Mixture separation selectivity.

## Data Availability

The data presented in this study are available upon request from the corresponding authors.
